# The Dynamic Change in the Neutrophil–Lymphocyte Ratio and Systemic Inflammatory Response Index After Undergoing an Intensive Resistance-Based Exercise Program

**DOI:** 10.3390/jfmk10040436

**Published:** 2025-11-08

**Authors:** Timothy P. Dougherty, David J. Carpenter, Chris Peluso, Colin E. Champ

**Affiliations:** 1 Department of Radiation Oncology, Allegheny Health Network, Pittsburgh, PA 15212, USA; colin.champ@ahn.org; 2 Allegheny Health Network Cancer Institute, Exercise Oncology and Resiliency Center, Pittsburgh, PA 15202, USA; 3 Department of Radiation Oncology, WellStar Paulding Medical Center, Hiram, GA 30141, USA

**Keywords:** breast cancer, exercise, resistance training, body composition, quality of life, survivorship, neutrophil–lymphocyte ratio, systemic inflammatory response index

## Abstract

**Background:** The change over time of certain inflammatory markers, such as the neutrophil–lymphocyte ratio (NLR) and systemic inflammatory response index (SIRI), is a prognostic factor in many cancers, including breast cancer. This study retrospectively evaluated how a 12-week intensive exercise program might have influenced both the NLR and SIRI in women with breast cancer. **Methods:** Two institutional review board-approved prospective clinical trials, EXERT-BC (NCT05747209, 2 November 2022) and EXERT-BCN (NCT05978960, 31 July 2023), were retrospectively assessed. Complete blood count (CBC) values performed before and after participation in a 12-week intensive resistance program were analyzed post hoc. Blood tests were ordered as part of routine clinical care and not pre-specified by either study protocol. Participants who had blood work more than four months from study intake or completion were excluded. Additionally, those undergoing active systemic therapy or with underlying inflammatory conditions were also excluded. The NLR and SIRI values were analyzed via the Mann–Whitney test, with pair-wise assessment of pre- and post-intervention values via the Wilcoxon signed-rank test. **Results:** Out of 84 participants, 21 people met the inclusion criteria. Roughly 70% had either ductal carcinoma in situ (DCIS) or early-stage breast cancer. The average blood draw was taken within two months of study intake and outtake. After the 12-week structured exercise program, there was an associated reduction in both the NLR (2.26 [IQR, 1.70–4.22] to 1.99 [1.44–2.62]; ΔNLR = −0.27, W = 47.0, *p* = 0.016) and SIRI (1.23 [0.82–1.64] to 0.80 [0.59–1.45]; ΔSIRI = −0.43, W = 48.0, *p* = 0.018). Of those who saw their inflammatory markers improve, roughly two thirds showed a clinically relevant improvement. **Conclusions:** Completion of a 12-week intensive resistance exercise program was associated with a statistically improved NLR and SIRI. The small sample size and retrospective nature limit the broader application of these findings. The results, however, provide a genesis for prospective validation examining the potential benefit exercise might have on the NLR and SIRI in women with breast cancer.

## 1. Introduction

Exercise is one of the most impactful activities for patients to improve their overall health and decrease mortality [1]. Recent data reveal that it may even improve outcomes after cancer treatment, including cancer-specific and all-cause mortality, and is associated with a reduction in local recurrence after the treatment of breast cancer [2,3,4]. Even with this information, however, exercise is not considered part of the standard of care for breast cancer patients, possibly due to the difficulty in encouraging physical activity as well as a lack of exercise infrastructure [5]. Additionally, it may also result from a lack of randomized evidence revealing an overall survival benefit. Only recently has a single phase III randomized trial shown that structured exercise improves disease-free survival and overall survival in those with locally advanced colon cancer [6]. For many other exercise-focused trials, however, there remains a difficulty in showing that exercise improves survival due to the smaller nature, and often lack of power, of these studies. Examining markers that serve as surrogates for survival, therefore, might circumvent this issue.

The neutrophil–lymphocyte ratio (NLR) reflects the internal cellular inflammatory state and is a prognostic metric when assessing individuals treated for cancer [7]. A similar measure, the systemic inflammatory response index (SIRI), which includes assessment of monocytes, also demonstrates good prognostication in a wide range of cancers [8]. The change over time of both the NLR and SIRI, defined as the dynamic change, is also a prognostic variable [9,10]. This latter attribute makes the dynamic change of the NLR and SIRI an attractive endpoint, since demonstrating a beneficial change in either of these metrics may decrease mortality. In other words, showing that an intervention, like exercise, can improve the NLR or SIRI might indirectly showcase an enhancement in more meaningful measures, such as overall survival. This is especially relevant when mortality data are difficult to measure or assess, particularly in trials where large recruitment is difficult, like exercise studies.

Although exercise tends to temporarily increase inflammatory markers post workout, consistent exercise appears to reduce inflammation [11]. The NLR appears to follow a similar pattern [12]. Exercise has been shown to reduce the neutrophil–lymphocyte ratio in overweight adolescents as well as in people with severe burns [13,14]. These results may be clinically relevant in conditions where chronic inflammation is an underlying component in the disease process, potentially explaining the prognostic ability of the NLR and SIRI with cancer outcomes. However, there is sparse evidence regarding the effect of exercise on NLR and SIRI in cancer patients, and particularly breast cancer patients.

Because consistent exercise appears to reduce inflammation, and because the NLR and SIRI are metrics of underlying inflammation, we hypothesized that the values of the NLR and SIRI would decrease after a 12-week exercise program. Due to the relationship between the NLR, SIRI, and their dynamic changes with cancer prognosis, we felt that this question might highlight the potential importance of surrogate markers for endpoints like overall survival in smaller trials. The goal of this project was to provide a foundation for future, prospective evaluation of the effect exercise might have on the NLR and SIRI, especially since these prognostic biomarkers are easy to calculate from routine blood tests.

## 2. Methods

We retrospectively assessed two institutional review board-approved prospective clinical trials registered at clinicaltrials.gov, EXERT-BC (NCT05747209, 2 November 2022) and EXERT-BCN (NCT05978960, 31 July 2023). Both protocols used an intensive 12-week regimen of resistance training incorporating multiple compound movements with sessions lasting for roughly 45 min, three days per week, and is described elsewhere [15,16]. In brief, the regimen, after a structured warm-up, consisted of push, pull, hip hinge, squat and core activation exercises with a target of approximately 10 sets per muscle group per week. Linear progressive overload was incorporated week over week to promote strength and hypertrophy. Supervising certified strength and conditioning specialists (CSCS) assessed repetition speed and rate of perceived exertion (RPE) to ensure safe dose escalation. Adherence—defined as attending >75% of sessions—was 94% (79 out of 84). Inclusion criteria for these prospective trials were women with breast cancer who could get up and down from the ground and squat their body weight. All participants signed informed consent.

From this group, and for this pilot trial, individuals with a complete blood count with differential (CBC w/diff) within four months prior to their enrollment and within four months after completion were evaluated post-hoc. Women who were undergoing systemic therapy concurrent with the trial or who had undergone systemic therapy within four months of enrollment were excluded. Additionally, participants were excluded if they received systemic therapy within three months of a CBC w/diff blood draw. Individuals with active inflammatory-like illnesses, including dermatologic, gastric, or other infections that may impact laboratory values, were excluded.

### Statistics

Descriptive statistics were used for those who demonstrated an increase versus a decrease in the NLR or SIRI. Continuous parameters were analyzed via the Mann–Whitney test, with pair-wise assessment of pre- and post-intervention values via the Wilcoxon signed-rank test. All statistics were performed using R (version 4.0.2, R Foundation for Statistical Computing, Vienna, Austria), with a *p*-value of <0.05 considered statistically significant.

## 3. Results

**Figure 1:**
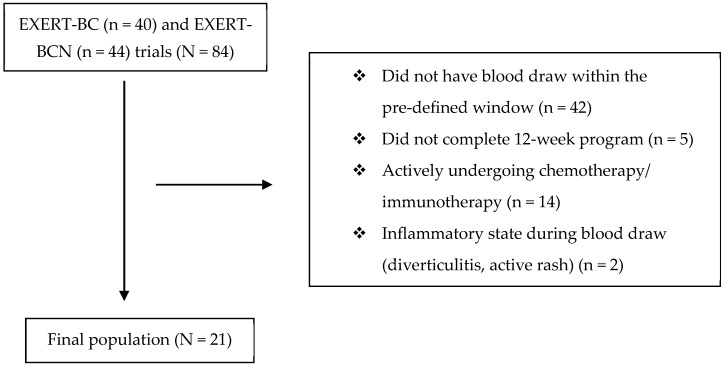
Consort diagram detailing the study population and reasons for exclusion.

In total, 84 participants enrolled in the EXERT-BC (n = 40) and EXERT-BCN (44) trials. Of these individuals, 23 met the inclusion criteria; however, two people were excluded due to active rash and diverticulitis, respectively, at the time of one of the blood draws, leaving 21 people for analysis (Figure 1). The average age of the cohort was 56. Most women (71%) had either ductal carcinoma in situ (DCIS) or early-stage breast cancer. Roughly one third received prior chemotherapy (38%), three-fourths received radiotherapy (76%), while two-thirds were actively on adjuvant endocrine therapy (62%). Of the sixteen women treated with radiation, three received their radiotherapy during the time of the trial, with the remaining 13 women completing radiation before the first CBC blood draw. None received chemotherapy during the exercise protocol. The average participant had a BMI of 30.3 kg/m^2^. Most individuals in this study had a CBC w/diff blood draw roughly two months prior to enrollment, and roughly two months after completion (Table 1).

**Figure 2:**
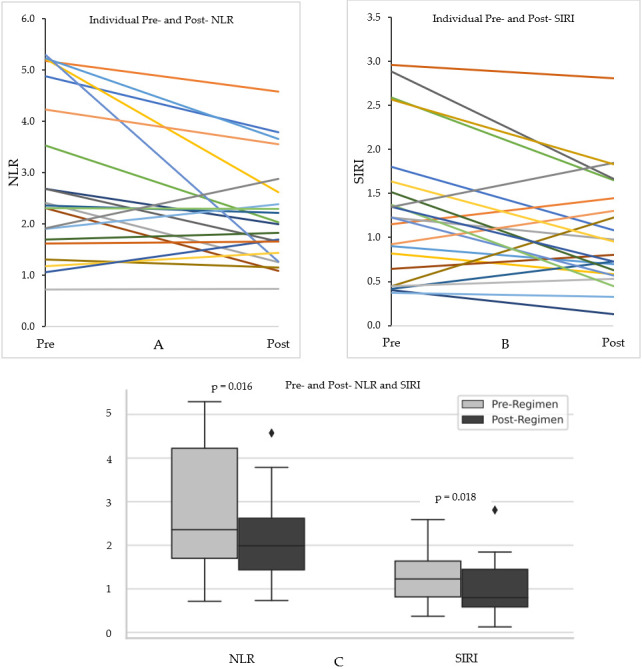
Pre- and post-exercise NLR and SIRI values before and after the 12-week intensive exercise regimen for different individuals (**A**,**B**) and the total population (**C**). Median NLR before the 12-week exercise program was 2.26 [IQR, 1.70–4.22] and 1.99 [1.44–2.62] after (ΔNLR = −0.27, W = 47.0, *p* = 0.016) (**C**). Median SIRI before the 12-week exercise program was 1.23 [IQR, 0.82–1.64] and 0.80 [0.59–1.45] after (ΔSIRI = −0.43, W = 48.0, *p* = 0.018) (**C**).

**Figure 3:**
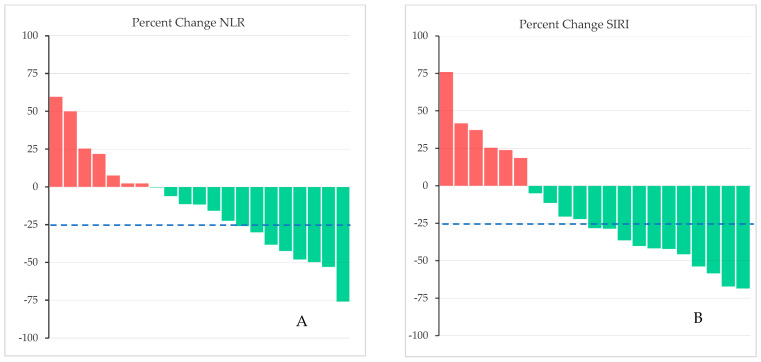
Percent change between pre- and post-exercise NLR (**A**) and SIRI (**B**) values. Green bars represent a decrease, whereas red bars denote an increase, in the respective ratio. Seven participants saw an increase in the NLR, whereas 14 experienced a decrease, after the 12-week intensive exercise regimen. Six participants saw an increase in the SIRI, whereas 15 experienced a decrease. Eight (38%) and eleven (52%) out of 21 experienced a greater than 25% decrease in the NLR and SIRI, respectively (dashed lines).

Pre-exercise NLR values were lower in those who ultimately saw an increase in the NLR or SIRI; however, the post-exercise NLR and SIRI values were not statistically different between those with an increase and those with a decrease in these metrics (Table 2). Figure 2A,B show individual pre- and post-exercise NLR and SIRI values, respectively. There was an associated reduction in both the NLR (2.26 [IQR, 1.70–4.22] to 1.99 [1.44–2.62]; ΔNLR = −0.27, W = 42.0, *p* = 0.016) and SIRI (1.23 [IQR, 0.82–1.64] to 0.80 [0.59–1.45]; ΔSIRI = −0.43, W = 48.0, *p* = 0.018) (Figure 2C). Of the 21 people evaluated, 15 (71%) had a decrease in the SIRI, whereas 6 (29%) had an increase (Figure 3B). Similar findings were seen for the NLR (Figure 3A). Of the 15 who experienced a decrease in the SIRI, 11 of them had a decrease of 25% or greater (dashed line, Figure 3B). Eight of 14 showed a decrease in the NLR by 25% or more (dashed line, Figure 3A).

## 4. Discussion

In this study, there was a statistically significant reduction between pre- and post-NLR and SIRI after completion of the three-month exercise program. To give broader clinical context to our results, although the optimal thresholds for the NLR and SIRI vary across studies, maintaining a low level (usually <2.0–2.2 for the NLR or <1.0–1.3 for the SIRI) or achieving at least a 25% reduction—particularly reductions exceeding 75%—have been associated with improved survival [17,18,19,20,21]. In this trial, median post-exercise values for NLR and SIRI were under 2.0 and 1.0, respectively, at the end of the 12-week exercise program. Additionally, just over half of the individuals evaluated (11 of the 21, 52%) showed a decrease in the SIRI by 25% or more. The lower final NLR and SIRI after the resistance training protocol, as seen in this pilot trial, might help explain one of the potential mechanisms by which exercise has been associated with decreased cancer mortality, namely via a reduction in inflammation—a known hallmark of cancer development and progression [22]. However, this interpretation is speculative, and our results must be interpreted with caution due to the small sample size, retrospective nature, and potential for confounding variables.

Those who did not experience an improvement in the NLR or SIRI tended to have lower baseline levels, suggesting that they may have already been in a low inflammatory state. A large review of the NLR in women with breast cancer revealed that comparison of the fourth quintile (NLR > 3.14) to the lowest quintile (NLR < 1.17) was associated with worse survival (HR = 1.27). However, little to no survival benefit was seen when comparing the third (or below) quintile (NLR < 2.26) to the lowest quintile (HR = 1.04) [23]. In our trial, of the 14 women who experienced a decrease in the NLR, 10 of them (71%) had values that decreased from the fourth to the third (or lower) quintile, suggesting that over two-thirds of participants may have had a clinically meaningful drop in their NLR. However, this claim is purely hypothesis-generating. In the group of people whose NLR increased, none demonstrated a rise from the third (or lower) quintile to the fourth. In other words, by the end of the exercise regimen, no one in the study had an NLR associated with worse survival.

Our results track with prior randomized data that show a decrease in the NLR after completion of an exercise routine in both obese adolescents and burn victims [13,14]. Additionally, two small randomized controlled trials looking at inflammatory markers in breast cancer survivors demonstrated that aerobic and resistance-based exercise programs reduced inflammatory markers, such as C-reactive protein (CRP) and tumor necrosis factor alpha (TNF-α), respectively [24,25]. These studies, however, did not report effects on the NLR or SIRI. Although our study was retrospective, it provides a foundation for randomized trials examining the potential impact of exercise on the NLR and SIRI. Such a trial would add value to the literature since the NLR and SIRI not only have good prognostic value, but are also widely accessible metrics from routine laboratory draws.

Although treating one’s cancer could theoretically reduce the inflammatory state described by the NLR and SIRI, most participants (18 of the 21) had completed their definitive chemotherapy and/or radiotherapy prior to the baseline CBC lab draw. Since the lab draws were part of routine clinical care and with the knowledge that the functional benefits of exercise tend to last even after completion of an exercise program [26], a several-month window is likely adequate in capturing changes in the NLR and SIRI that might be attributable to the exercise program. We acknowledge, however, that this is an important assumption. This study is the first of its kind to retrospectively evaluate the dynamic change in the NLR and SIRI after a 12-week intensive exercise program in women with breast cancer.

### Limitations

Several limitations are present in this study. Firstly, it was a retrospective secondary analysis of two prospective studies with a small sample size, which lacked a control group and randomization. The fact that lab draws were taken at different time points in relation to the start and end of the study may have also led to variability in results. Additionally, the blood draws were performed as part of routine clinical care and were not ordered specifically for analyzing the dynamic change that an exercise program might have on the NLR and SIRI. As such, selection bias could have occurred, with our included population (people receiving routine bloodwork) potentially differing from those excluded (people not receiving routine bloodwork). We acknowledge that the relatively large time window (within 4 months) for extracting CBC data might have allowed factors other than the exercise routine to have influenced the values of the blood draws. Due to the small sample size and lack of randomization, regression to the mean might also explain our results. Lastly, we could not account for possible confounders, such as diet, medication use, treatment type, time from treatment, and activity outside the program, among other potential confounding variables. Multiple hypothesis testing is another limitation. Due to the correlation of the NLR and SIRI with each other as well as the exploratory nature of this project, we did not perform multiple hypothesis correction. Overall, all these shortcomings limit the conclusions we can draw from this study. This pilot trial, however, aimed to provide impetus for prospective evaluation of the potential impact an exercise program might have on the NLR and/or SIRI.

## 5. Conclusions

A 12-week, resistance-based exercise program was associated with an improved NLR and SIRI, suggesting that an exercise program might promote lower levels of inflammation; however, this conjecture is speculative given the small, non-randomized, and retrospective nature of this pilot trial. Further research assessing the NLR and SIRI as surrogate endpoints for survival is warranted, particularly for smaller trials and exercise interventions.

## Figures and Tables

**Table 1:** Characteristics of the Study Population.

Characteristic	Total Population (N = 21)	Those with an Increase in the SIRI (n = 6)	Those with a Decrease in the SIRI (n = 15)
Age (yrs) [range]	56 [40–69]	52 [40–58]	57 [42–69]
Race			
White	18 (86%)	5 (83%)	13 (87%)
Black	3 (14%)	1 (17%)	2 (13%)
Stage			
0	2 (10%)	1 (17%)	1 (7%)
1	13 (61%)	4 (67%)	9 (60%)
2	4 (19%)	0 (0%)	4 (27%)
3	2 (10%)	1 (17%)	1 (7%)
Prior Chemotherapy (%)	8 (38%)	2 (33%)	6 (40%)
Received Radiotherapy (%)	16 (76%)	3 (50%)	13 (87%)
Current adjuvant endocrine therapy (%)	13 (62%)	4 (67%)	9 (60%)
BMI (kg/m^2^) [IQR]	30.3 [25.6–35.6]	31.5 [27.4–34.9]	29.8 [25.3–34.5]
Time between initial CBC and intake (days) [IQR]	64 [26–103]	86 [73–106]	55 [14–91]
Time after outtake and final CBC (days) [IQR]	57 [18–79]	56 [16–101]	57 [25–76]

**Table 2:** Median pre- and post-exercise NLR and SIRI values.

	Total Population (N = 21)	Those with an Increase in the SIRI (n = 6)	Those with a Decrease in the SIRI (n = 15)	Mann-Whitney *p*-Value
Pre-exercise NLR [IQR]	2.26 [1.70–4.22]	1.76 [1.29–1.92]	2.69 [2.33–5.03]	0.016
Pre-exercise SIRI [IQR]	1.23 [0.82–1.64]	0.78 [0.50–1.10]	1.37 [1.06–2.18]	0.061
Post-exercise NLR [IQR]	1.99 [1.44–2.62]	1.99 [1.66–2.36]	1.99 [1.26–3.09]	0.904
Post-exercise SIRI [IQR]	0.80 [0.59–1.45]	1.05 [0.75–1.41]	0.73 [0.57–1.36]	0.484

## Data Availability

The original contributions presented in this study are included in the article. Further inquiries can be directed to the corresponding author.
